# The Cellular and Organismal Effects of Nitroxides and Nitroxide-Containing Nanoparticles

**DOI:** 10.3390/ijms25031446

**Published:** 2024-01-24

**Authors:** Izabela Sadowska-Bartosz, Grzegorz Bartosz

**Affiliations:** Laboratory of Analytical Biochemistry, Institute of Food Technology and Nutrition, College of Natural Sciences, University of Rzeszow, 4 Zelwerowicza Street, 35-601 Rzeszow, Poland; gnartosz@ur.edu.pl

**Keywords:** nitroxides, TEMPO, TEMPOL, TEMPAMINE, redox nanoparticles

## Abstract

Nitroxides are stable free radicals that have antioxidant properties. They react with many types of radicals, including alkyl and peroxyl radicals. They act as mimics of superoxide dismutase and stimulate the catalase activity of hemoproteins. In some situations, they may exhibit pro-oxidant activity, mainly due to the formation of oxoammonium cations as products of their oxidation. In this review, the cellular effects of nitroxides and their effects in animal experiments and clinical trials are discussed, including the beneficial effects in various pathological situations involving oxidative stress, protective effects against UV and ionizing radiation, and prolongation of the life span of cancer-prone mice. Nitroxides were used as active components of various types of nanoparticles. The application of these nanoparticles in cellular and animal experiments is also discussed.

## 1. Introduction

Redox equilibrium is an important element of the functioning of cells and organisms. This somewhat vague term covers an equilibrium between the intensity of undesired side effects of oxidative processes and the activity of antioxidants, or rather a set of dynamic equilibria, different in various cellular compartments and changing depending on the functional state of the cell and the organism. Antioxidants are important players in this dynamic equilibrium. There are numerous antioxidants in the body produced endogenously and ingested in food from external sources. One may wonder if there is any benefit to using synthetic antioxidants not occurring in nature. Nevertheless, the plethora of fully synthetic drugs, with no natural counterparts, are quite effective in treating various diseases, and synthetic antioxidants with new properties may also prove quite useful.

One group of synthetic antioxidants is nitroxides. They are unusual in that they are stable free radicals, with odd electrons on the nitroxyl N-O^•^ group, surrounded by bulky methyl or ethyl groups, thereby considerably limiting the reactivity of the nitroxyl group and conditioning its considerable stability. Nitroxides have found a wide range of applications in biology and medicine. They have been used to monitor intracellular redox reactions, oxygen concentration, and pH, as contrast agents in magnetic resonance imaging, and as probes in EPR imaging. The main biomedical applications of nitroxides are due to their antioxidant properties. Nitroxide has some disadvantages, mainly the short lifetime in the body. To overcome these limitations, several types of nanoparticles containing nitroxide residues have been synthesized and studied. This review is devoted to the antioxidant action of nitroxides and nitroxide-containing nanoparticles in cellular and organismal systems.

## 2. Structure of Nitroxides

**Figure 1:**
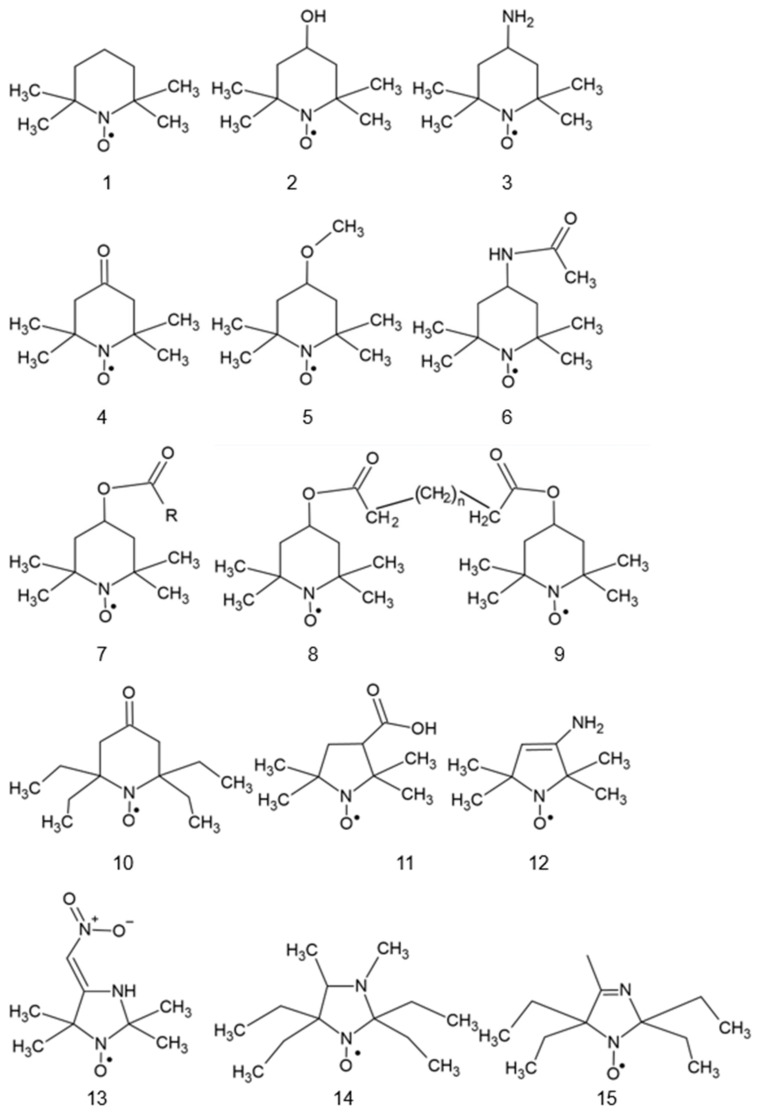

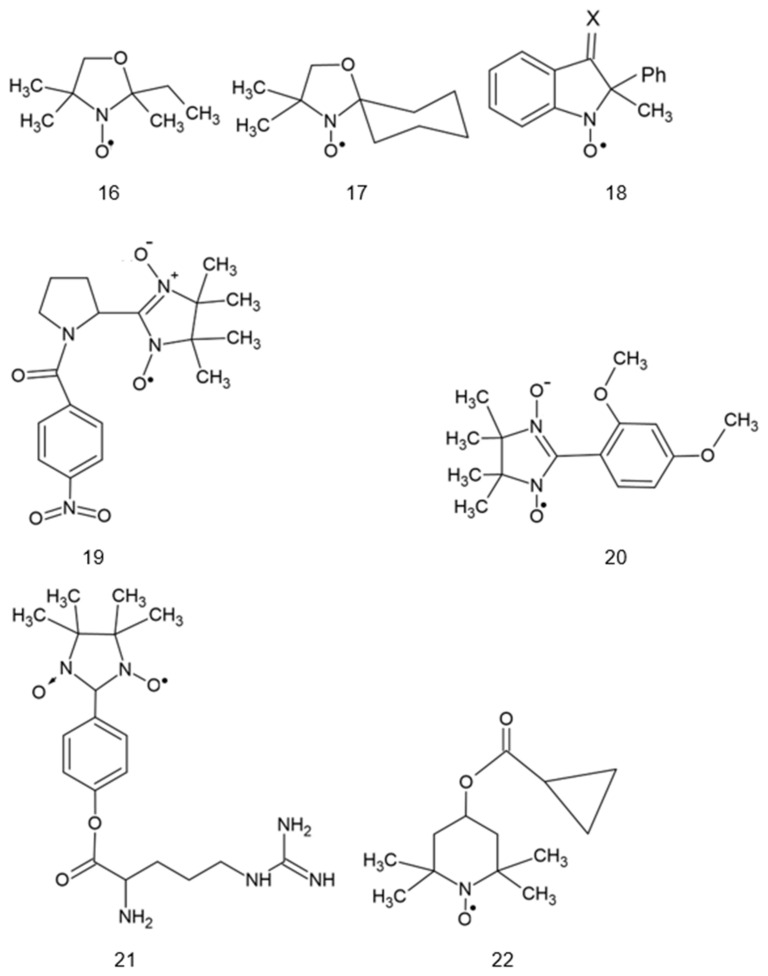

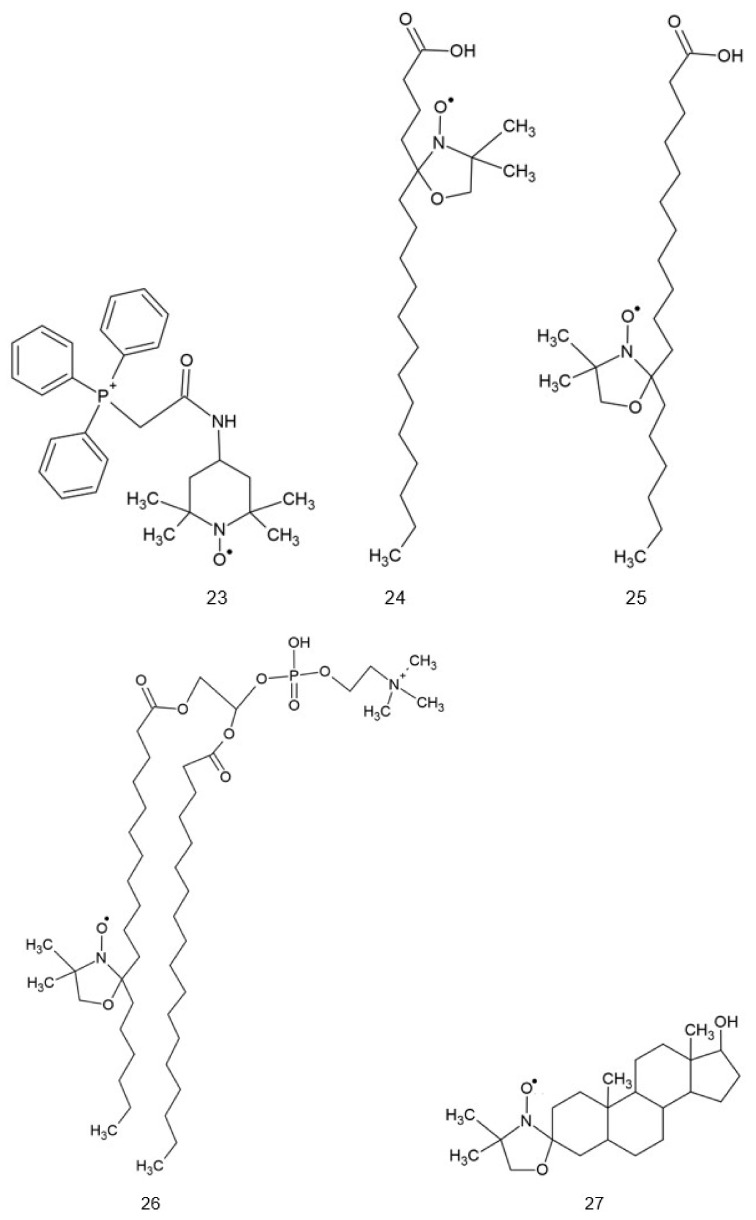
Structures of various nitroxides. 1: 2,2,6,6-tetramethylpiperidine-N-oxyl (TEMPO); 2: 4-hydroxy-TEMPO (TEMPOL); 3: 4-amino-TEMPO (TEMPAMINE); 4: TEMPONE; 5: 4-methoxy-TEMPO; 6: 4-acetamido-TEMPO; 7: TEMP: TEMP2, R = CH_3_CH_2_; TEMP8, R = (CH_2_)_7_CH_3_; 8: TINO; 9: TEEPONE; 10: 3-carboxy-PROXYL (3-CP); 11: 4-amino-2,2,5,5-tetramethyl-3-imidazoline-1-oxyl (ATI); 12: 3-carbamoyl-proxyl (3-CP); 13: 2,2,5,5-tetramethyl-4-nitromethylene-3-imidazolidine-N-oxyl (NTI); 14: 3,4-dimethyl-2,2,5,5-tetraethylperhydroimidazol-1-oxyl); 15: 4-methyl-2,2,5,5-tetraethyl-2,5-dihydro-1H-imidazol-1-oxyl; 16: OXANO; 17: CHD; 18: 1,2-dihydro-2-methyl-2-phenyl-3H-indole-3-phenylimino-1-oxyl (X = NPh or = CH_2_CH_3_); 19: L-N-*p*-nitrobenzoylpyrrolidinyl(4,5-dihydro-4,4,5,5-tetramethyl-3-oxido-1H-imidazol-3-ium-1-oxyl-2-yl) (L-NNNBP); 20: (2,4-dimetoxyphenyl)-4,4,5,5-tetra-methylimidazoline-1-oxyl-3-oxide (NNR); 21: NNR-arginine conjugate; 22: OT-551; 23: 2-(2,2,6,6-tetramethylpiperidin-1-oxyl-4-ylamino)-2-oxoethyl)triphenylphosphonium (Mito-TEMPO); 24: 5-doxylstearic acid; 25: 16-doxylstearic acid; 26: lecithin labeled with 12-doxylstearic acid; 27: 3-doxylcholestane.

Most nitroxides are derivatives of substituted derivatives of pyrrolidinyl-N-oxyl (6-membered ring) or pyrrolidinyl-N-oxyl (5-membered ring). The structures of the most used nitroxides and some others mentioned in this paper are presented in Figure 1.

Some nitroxides may be targeted to specific sites in the cell. The coupling of antioxidants to a membrane-permeable cation targets them to mitochondria as these organelles, with their inner membrane potential of −180 V, are the most electronegative site in the cell. Thus, synthesized mito-TEMPO (Figure 1, #23) accumulates in the mitochondria and and may exert its antioxidant action at the main site of superoxide generation in the cell [1,2].

Spin-labeled fatty acids, cholestane, and phospholipids (Figure 1, #24–27) locate in cell membranes like their natural precursors [3,4].

## 3. Reactivity of Nitroxides

Nitroxides are known to enter one-electron reactions with other radicals, oxidize transition metal ions, and stimulate the catalase-like activity of hemoproteins. Nitroxides (both indolinonic and piperidine) effectively scavenge alkyl, aroyloxyl [5], phenoxyl [6], alkoxyl, and peroxyl radicals [7,8].

Nitroxides react rapidly with alkyl and peroxyl radicals. The reaction of TEMPO with alkyl radicals occurs with diffusion-limited rates, with rate constants of ~1–3 × 10^10^ M^−1^ s^−1^ (for TEMPO), thereby enabling this compound to compete with O_2_ for alkyl radicals [9]. The reactions may consist of electron or hydrogen atom transfer or the addition of a carbon-centered radical to the nitroxide:

R′ + R-N-O^●^ → R-N-O-R′

where R′ is a carbon-centered radical [10].

Genovese et al. [11] compared the reaction rate constants of several TEMPO analogs with peroxyl radicals generated by the thermal decomposition of 4,4′-azobis(4-cyanovaleric acid) (ABCV) and subsequent reaction of so-formed alkyl radicals with oxygen. Their results, as shown in Table 1, exhibit a correlation between the reaction rate constant of the nitroxides studied with peroxyl radicals and their redox potentials. Although the values of the standard redox potentials determined by various authors differ [11,12,13], the effect of substituents on these values is consistent.

Nitroxides are efficient scavengers of peroxyl radicals, so they can be expected to inhibit lipid peroxidation and other reactions induced by peroxyl radicals. TEMPOL protected against the AAPH-induced DNA breakage and the AAPH-induced inactivation of glucose oxidase [14]. TEMPO inhibited lipid peroxidation in soybean phosphatidylcholine liposomes [15]. Carbonylation of bovine serum albumin (BSA) was observed upon UVA illumination in the presence of Parsol 1789, forming free radicals upon irradiation (initially carbon-centered, and then oxygen-centered), apparently due to the scavenging of these radicals. TEMPO and TEMPOL inhibited BSA carbonylation [16]. Lipid peroxidation in phosphatidylcholine liposomes induced by UVA in the presence of Parsol 1789 was inhibited by nitroxides, the order of protection efficiency being: TINO (Figure 1, #8) > TEMP8 > TEMP2 (Figure 1, #7) > hydroxylamine of TINO [17]. Nitroxide radicals (TEMPO, TEMPOL, and indolinonic nitroxides) protected against strand breaks inflicted on DNA when illuminated in vitro in the presence of dibenzoylmethane and Parsol 1789, used as a UVA-absorbing sunscreen. In contrast, vitamin E had no protective effect under the conditions used. These results might have practical implications because of the widespread use of vitamin E as an antioxidant in cosmetics, including sunscreens [10]. When the efficiency of a series of indolinic nitroxides of different lengths of the hydrophobic tail to protect against protein oxidation and lipid peroxidation were compared, 2-dihydro-2-decyl-2-phenyl-3H-indole-3-phenylimino-1-oxyl (Figure 1, #18, R = (CH_2_)_9_CH_3_)) was the most effective in the inhibition of carbonyl formation of BSA but 1,2-dihydro-2-ethyl-2-phenyl-3H-indole-3-phenylimino-1-oxyl) (R = CH_2_CH_3_) was the most effective in preventing malondialdehyde formation in rat liver microsomes [18].

Nitroxides are efficient scavengers of ^•^NO_2_ at physiological pH (k = (3–9) × 10^8^ M^−1^ s^−1^) and belong to the most effective metal-independent scavengers of CO_3_^•−^ radicals (k = (2–6) × 10^8^ M^−1^ s^−1^) [19].

Nitroxides also react with peroxynitrite. TEMPOL was found to inhibit the peroxynitrite-mediated nitration of phenolic compounds in the presence of a large molar excess of peroxynitrite over the pH range of 6.5–8.5, suggesting a catalytic-like mechanism of peroxynitrite decomposition. In these experiments, inhibition was specific for nitration and did not affect hydroxylation [20]. TEMPOL was oxidized by peroxynitrite-derived radicals (^•^OH and CO_3_^•−^, in the absence and presence of carbon dioxide, respectively) to the oxoammonium cation, which, in turn, was reduced back to TEMPOL while oxidizing peroxynitrite to oxygen and nitric oxide [21]. However, no correlation was established between the kinetics of nitroxide reactions with HO_2_^•^/O_2_^•−^, ^•^NO_2,_ and CO_3_^•−^ and their protective activity against biological oxidative stress [19].

The EPR signal of nitroxides in aqueous solutions is destroyed by ionizing radiation, mainly due to oxidation to oxoammonium cation by the hydroxyl radical; however, the oxoammonium cation can be reduced back by the hydrated electrons of H atoms [22].

Nitroxides, like nitric oxide, are efficient scavengers of protein radicals generated via radical transfer from H_2_O_2_-activated horseradish peroxidase, radicals formed on myoglobin via reaction with H_2_O_2_, and carbon-centered radicals formed from amino acid hydroperoxides on exposure to Fe^2+^-EDTA [23]. Nitroxides are considered sterically hindered, structural mimics of NO^●^ because both types of compounds contain an odd electron, which is delocalized over the nitrogen-oxygen bond. The biological activity of nitroxides is often attributed to their NO^●^-mimetic properties. Nitroxides are more convenient in such applications since they are mostly air-stable crystalline solids, in contrast to NO^●^, which is unstable and gaseous at room temperature [24].

From a biological point of view, the reactions of nitroxides with the superoxide radical anion are especially interesting. The exposure of five-membered (pyrrolidinyl) nitroxides to superoxide flux results in a decrease in their EPR signal. The signal loss is reversed by the addition of ferricyanide, indicating that superoxide reduces the nitroxide to its respective hydroxylamine [25]. One-electron reduction in stable nitroxides to the corresponding hydroxylamine was proposed to be the primary metabolic pathway [26]. A similar loss of EPR signal was not found for six-membered ring piperidine nitroxides (TEMPO, TEMPOL, and TEMPAMINE); the effect was ascribed to the facile reoxidation of the corresponding hydroxylamine by superoxide [27]. However, the reaction rate of the corresponding hydroxylamines with superoxide is slow; for Tempol-H it was found to be 4 × 10^2^ M^−1^ s^−1^ [26]. This value is two orders of magnitude lower than that estimated for the reaction of superoxide with TEMPOL and in the same order of magnitude estimated for TEMPO [28]. On this basis, Krishna et al. concluded that the previously proposed mechanism of superoxide dismutation involving hydroxylamine as an intermediate is not consistent with these observed rates [26]. They proposed that the initial reaction with superoxide is a one-electron oxidation to the oxoammonium cation. Piperidine derivatives such as TEMPO and TEMPOL are readily oxidized by protonated superoxide (perhydroxyl radical) ^●^OOH to yield oxoammonium cation. The oxoammonium cation may either be reduced back to the nitroxide by superoxide or may react with NADH or NADPH to form the corresponding hydroxylamine [26]. During the dismutation of superoxide, high steady-state levels of piperidinyl and proxyl derivative nitroxides are maintained. This suggests that the reduction in the oxoammonium cation by superoxide is relatively fast. The O_2_^●−^/H_2_O_2_ couple has a redox potential of +0.89 V (although a value of 1.06 was also reported) [29], so the oxidation rather than reduction in nitroxides is to be expected. The doxyl derivatives, on the other hand, are directly reduced by superoxide to their hydroxylamines [26,27]. The following reaction cycle was proposed [30]:

RR′NO^●^ + O_2_^●−^ + 2 H^+^ → RR′NO^+^ + H_2_O_2_ (1)

RR′NO^+^ + O_2_^●−^ → RR′NO^●^ + O_2_ (2)

The catalytic rate constants for O_2_^●−^ dismutation, determined for TEMPO and TEMPOL, were found to increase with decreasing pH, indicating that ^●^OOH rather than O_2_^●−^ is oxidizing the nitroxide [30]. Direct evidence for the reaction of the oxoammonium cation with superoxide (reaction 2) was not obtained, but in biological systems, this intermediate may be expected to react with many endogenous one- and two-electron reducing agents. Thus, the pathway for superoxide dismutation in vivo would not necessarily involve direct reduction of O_2_^●−^ (reaction 2) as the major route for regeneration of the cyclic nitroxide. Given the instability of the oxoammonium cation, it would appear that regeneration of the nitroxide would occur predominantly by reaction of the oxoammonium with endogenous substrates other than superoxide. This raises the possibility of deleterious reactions with critical biomolecules if repair (re-reduction) mechanisms are too slow to prevent subsequent irreversible processes [26].

The cellular destruction of persistent spin adduct nitroxides may be facilitated by primary univalent oxidation. The reactions of nitroxides with superoxide reveal that the piperidinyl and the proxyl derivatives were reduced in a superoxide-dependent manner only in the presence of two-electron donors such as NADH and NADPH. The reduction products of these nitroxides are the corresponding hydroxylamines. These results suggest that while the initial reaction with superoxide is one-electron oxidation to the oxoammonium cation, this transient species either may be reduced back to the nitroxide by superoxide or may react with NADH or NADPH to form the corresponding hydroxylamine [26]. Superoxide was demonstrated to reduce nitroxides to their corresponding hydroxylamines in the presence of sulfhydryl-containing compounds (3 nitroxides were reduced per superoxide). Superoxide directly reacts with nitroxide to yield a *N*-hydroxy-*N*-hydroperoxyl compound. This product rapidly decomposes, giving a hydroxylamine and an oxidized sulfhydryl compound, postulated to be a sulfenyl hydroperoxide. It was hypothesized that this sulfenyl hydroperoxide reduces two additional nitroxyl free radicals to account for the unusual stoichiometry [31].

Thus, nitroxides catalytically decompose superoxide, showing a pseudoenzymatic superoxide dismutase activity and are superoxide dismutase mimics. They are less efficient than superoxide dismutases, which dismutate superoxide with second-order rate constants exceeding 10^9^ s^−1^M^−1^ [32] but are usually membrane-permeable and thus act intracellularly. The therapeutic application of superoxide dismutase (SOD) has been proposed, but exogenously added SOD may be immunogenic, does not penetrate readily into the cells, and unless bound to a protein or polyethylene glycol, has a metabolic half-life of only several minutes as a small protein [33]. Therefore, cell-permeable, low-molecular-weight compounds that mimic the activity of SOD were also proposed. Usually, SOD mimics are chelates of transition metals such as copper, iron, or manganese, which, like the metal center in native SOD, can undergo alternate reduction and oxidation. However, metal-containing SOD mimics are prone to dissociation in the presence of cellular proteins, thus not only causing loss of SOD mimic activity but also mobilization of potentially toxic redox-active metals [30,34]. In contrast, nitroxides do not release metal ions and even oxidize transition metal ions, preventing their participation in the Fenton reaction.

The catalytic dismutation rates were found to be directly related to the midpoint redox potential of the nitroxides. The catalytic rates of O^●−^_2_ dismutation for TEMPO. TEMPOL and TEMPAMINE were characterized by rate constants of 1.2 × 10^5^ M^−1^ s^−1^, 6.5 × 10^4^ M^−1^ s^−1^, and 6.5 × 10^4^ M^−1^ s^−1^, respectively [26], although higher values of superoxide dismutation by nitroxides, up to 10^6^ M^−1^ s^−1^ to 10^8^ M^−1^ s^−1^ were also reported [35].

Nitroxides were found to stimulate the decomposition of hydrogen peroxide by heme proteins. By shuttling between two oxidation states (nitroxide and oxoammonium cation), stable nitroxides (R-NO^●^) enhance the catalase-mimetic activity of myoglobin (Mb) by reducing MbFeIV to MbFeIII, thus facilitating H_2_O_2_ dismutation accompanied by oxygen evolution.

MbFeIV + R-NO^●^ → MbFe III + R-NO^+^ (3)

The oxoammonium species has indeed been isolated as an end product in the hemoglobin/nitroxide system upon the addition of hydrogen peroxide [36]. Myoglobin is more readily activated than oxymyoglobin to the ferryl states, which are strong oxidants capable of inflicting significant damage by oxidizing a number of biological targets, including initiation of lipid peroxidation in biological membranes [37]. Generally, reagents reducing MbFeIV to MbFeIII operate in a stoichiometric manner; nitroxide radicals, which shuttle among three oxidation states, can detoxify hypervalent metals in a catalytic fashion and provide protection against hypervalent heme iron.

The oxoammonium cation R-NO^+^ formed (3) can oxidize another H_2_O_2_ molecule [34]:

H_2_O_2_ + R-NO^+^ → R-NO + O_2_^●−^ + 2 H^+^ (4)

## 4. Reduction of Nitroxides

The chemical reduction of nitroxides to EPR-silent hydroxylamines, in many cases, is an unfavorable factor that significantly limits their applications in biological systems. On the other side, EPR-measured rates of nitroxide reduction have been shown to provide information on tissue redox status [38,39,40], and reactive oxygen species (ROS) generation in vivo. The enhanced generation of hydroxyl radicals accelerated the disappearance rate of the EPR signal of nitroxides [41].

In erythrocytes and many other cell types, ascorbate is the predominant component responsible for the reduction of nitroxides [42].

The reduction rates of NR by ascorbate normally correlate with its electrochemical reduction potential and depend on the nature of the radical ring, charge of the radical, and steric shielding of nitroxyl fragment [43]. The bimolecular rate constants of ascorbate-induced reduction are significantly higher for six-membered ring nitroxide of piperidine types (e.g., 3.5 M^−1^ s^−1^ for TEMPO [44] and 7 M^−1^ s^−1^ for TEMPOL [45] than for the five-membered ring nitroxides of pyrrolidine (0.07–0.3 M^−1^ s^−1^) [43,44] and imidazolidine (0.85 M^−1^ s^−1^) [43] types. The presence of the double bond at position 3 in the five-membered ring nitroxides of pyrroline and imidazoline types increases their reduction rates by ascorbate. A negative charge is a factor stabilizing NR against reduction by the negatively charged ascorbate anion [43]. The reduction rate of 4-methyl-2,2,5,5-tetraethyl-2,5-dihydro-1H-imidazol-1-oxyl by ascorbate was much lower than that of 3-carboxy-2,2,5,5-tetramethyl-1-pyrrolidine-1-oxyl. A tetraethyl-substituted imidazole nitroxide (Figure 1, #14) [46] was characterized by the lowest rate constant for the reaction with ascorbate (0.02 M^−1^ s^−1^) [43].

The reaction of nitroxides with glutathione (GSH) is of particular interest due to the importance of GSH in the regulation of intracellular redox status. The appreciable chemical reduction of nitroxides via GSH does not occur over a few hours [31,47,48]. However, GSH can significantly contribute to the reduction of nitroxides in biological systems indirectly by acting as a secondary source of reducing equivalents [39]. In the presence of ascorbate, the addition of GSH facilitated the reduction of nitroxides by ascorbate, which was attributed to the scavenging of the ascorbate radical by GSH and inhibition of oxidation of the hydroxylamine formed by the ascorbate radical Asc^•−^ formed during nitroxide reduction to hydroxylamine.

Ascorbic acid and reduced glutathione, as well as both compounds together, reduced piperidine nitroxides to corresponding hydroxylamines exclusively. Neither corresponding secondary amines were found [49]. Different substituents at the C2 and C4 positions affect the susceptibility of piperidine nitroxides toward ascorbate reduction [50]. In contrast to methyl-substituted nitroxides, tetraethyl-substituted piperidine nitroxides bearing an exocyclic double bond like TEEPONE (Figure 1, #9) are also substrates to fast P450-induced hydrogen atom abstraction in α-position to the sp^2^ carbon of the ring, followed by the destruction in vivo [51].

NADH is an obligatory two-electron reductant. No direct reaction of piperidine nitroxides with NADH was observed [26]. In human keratinocytes isolated from foreskin and breast skin, acetamido-2,2,6,6-tetramethylpiperidine-*N*-oxyl benzyl dimethylammonium bromide (spin-labeled quat) was reduced by thioredoxin reductase localized at the outer plasma membrane but not by glutathione reductase. The reduction was inhibited by pCMB, DTNB, and NADP^+^, inhibitors of thioredoxin reductase [52]. Five-membered ring nitroxides and α-carboxy α-aryl *tert*-butyl nitroxides were more resistant toward reduction by liver homogenates than six-membered ring and heterocyclic-substituted nitroxides in all systems. The presence of carboxylate groups tends to enhance the resistance of all the nitroxides toward reduction by liver homogenate, hepatocytes, and subcellular fractions [53]. In the keratinocytes, TEMPO was reduced to hydroxylamine and secondary amine, while for TEMPAMINE, the sole detected metabolite was the corresponding hydroxylamine. No evidence for the formation of glucuronides or sulfates was found in the keratinocyte cell line HaCaT. The reduction of the hydroxylamine was only partially thiol-dependent in keratinocytes. The lost ESR signal of TEMPO was recovered upon the addition of the mild oxidant ferricyanide to 70–80% [49].

In photosynthesizing cells, nitroxides are also reduced by the photosynthetic apparatus. The rate of nitroxide reduction upon illumination of PS II depends on the chemical structure of radicals and the capability of their coming close to low-potential redox centers of photoactive PS II complexes. Nitroxide radicals NTI (2,2,5,5-tetramethyl-4-nitromethylene-3-imidazo-lidine-N-oxyl) and TACET (4-hydroxy-2,2,6,6-tetramethylpiperidine-1-oxyl-acetate; Figure 1 #7, R = CH_3_), containing polar groups, appear to be most efficient acceptors of electrons donated by PS II compared to neutral (TEMPOL) or positively charged (TEMPAMINE) spin labels [54].

Hydrophobic 5-doxyl stearate (Figure 1, #24) localizes to cellular membranes and, in normal erythrocytes, is not reduced, in contrast to TEMPO, TEMPOL or TEMPONE, located in the aqueous phase. However, in erythrocytes infected with the malarial parasite *Plasmodium berghei*, it is subject to reduction; this effect was ascribed to increased oxidative stress in these cells [55]. Similar behavior can be expected in other cases of intense oxidative stress.

## 5. Cellular Effects of Nitroxides

Numerous experiments demonstrated various beneficial antioxidant effects of nitroxides, i.e., protection of cells against oxidative stress induced by various factors (some of them as models of clinically relevant situations, e.g., ischemia reoxygenation [56], UV [57], and ionizing radiation [58,59]). Protective effects of nitroxides were also demonstrated in neuroblastoma cells treated with 6-hydroxydopamine (a model of Parkinson’s disease) [60,61,62], cells treated with high concentrations of glucose (a model of diabetes) [63,64], spermatozoa [65,66,67], and oocytes [68].

TEMPOL was found to not be mutagenic and to afford protection against mutagenic effects [69,70,71]. However, nitroxides were also reported to be mutagenic using the *Salmonella typhimurium* mutagenicity test. Nitroxide mutagenicity was dramatically increased in the presence of the superoxide radical generating system, xanthine oxidase/hypoxanthine [70].

Among the six nitroxides tested, 3-aminomethyl-PROXYL and TEMPAMINE were the most effective in protecting V79 hamster cells against ionizing radiation. These nitroxides possess amine groups which, at intracellular pH, would be partly positively charged, thus allowing for their association with negatively charged DNA. The radioprotection was hypothesized to be conditioned, at least in part, to the ability of the nitroxides to associate with DNA. Indeed, these nitroxides showed the highest extent of association with DNA in comparison with other nitroxides [59].

Coronavirus 2 (SARS-CoV-2), the causal agent of COVID-19, uses an RNA-dependent RNA polymerase (RdRp) for the replication of its genome and the transcription of its genes. The catalytic subunit of the RdRp, nsp12, ligates two iron–sulfur metal cofactors in sites that were modeled as zinc centers of the RdRp complex. These metal binding sites are essential for replication and interaction with viral helicase. Oxidation of the clusters by the stable nitroxide TEMPOL caused their disassembly, potently inhibited the RdRp and blocked SARS-CoV-2 replication in cell culture [72].

Interestingly, in some situations, the protective effects of products of nitroxide reduction, hydroxylamines, were demonstrated. TEMPOL hydroxylamine (OT-674) showed desirable safe and strong protective activities against oxidative damage in the ocular tissue. OT-674 was shown to be an effective singlet oxygen quencher. Pretreatment protected light-irradiated ARPE-19 cells that had accumulated A2E chromophore [73]. However, OT-674 is unable to effectively cross the cornea and is not feasible for applications to the back of the eye. Therefore, it was chemically modified into a prodrug, OT-551 (with a cyclopropyl group and an ester linkage), which is more lipophilic and fully capable of penetrating the cornea and can travel via the scleral route to reach the macula at the back of the eye. In the eye, ocular esterases can convert OT-551 to more water-soluble, less lipophilic, TEMPOL hydroxylamine, which can function in the back of the eye, at the macula and retina, and be helpful in preventing age-related macular degeneration (AMD). OT-551, formulated as a topical daily eye drop for dry AMD patients, is capable of significant preservation of both standard luminance and low luminance visual acuity [74].

An Interesting distant effect of TEMPO was reported due to the volatility of this compound, which is rather unique among antioxidants. Concentration-dependent inhibition of ferroptosis on human fibrosarcoma cells by TEMPO present in a neighbor vessel was reported [75]. This result suggests a possibility of administration of TEMPO in the form of vapor.

Various cellular effects of nitroxides are exemplified in Table 2.

Adverse cellular effects of nitroxides were also reported, attributable mainly to the pro-oxidant action of the oxyammonium cations. Nitroxides, like any compounds, are cytotoxic at appropriately high concentrations. Toxicities of five nitroxides for human HaCaT keratinocytes correlated with the lipid/water partition coefficients of the investigated nitroxides, with IC_50_ values ranging from 1.1 mM (TEMPOL-benzoate) to 11.1 mM (TEMPOL) [49]. These effects cannot always be conceived as deleterious. The activation of Nrf2 by nitroxides (probably mainly by the oxoammonium cations) may be another mechanism of their antioxidant action at the cellular level [91]. Similarly, cytotoxicity to malignant cells or microbial pathogens is beneficial for the organism. Some adverse cellular effects of nitroxides are presented in Table 3.

## 6. Effects of Nitroxides in Animal Experiments

Numerous experiments demonstrated the effects of nitroxides in experimental animals. Listing all of them would be too cruel for the readers, so only a selection is presented in Table 4. A comprehensive review of early experiments concerning the protective effects of nitroxides in oxidative stress and their hypotensive action is presented in reviews by Wilcox and Pearlman [101] and Wilcox [102], while their action on cancer cells was reviewed more recently by Lewandowski and Gwożdziński [103].

For TEMPOL injection at a dose of 250 mg/kg, the peak whole blood concentration of TEMPOL was about 600 μg/mL (about 3.5 mM) 5–10 min after injection [59]. Tempol-administered animals in the feed consumed ca. 20–30% less food than control animals, but it did not seem to be due to a lower attractiveness of TEMPOL-containing food. The leptin levels were also significantly (by about one-half) lower in TEMPOL-treated animals when compared to control groups. Increased levels of the uncoupling protein 2 in the skeletal muscle might also contribute to weight loss [104]. For TEMPOL hydroxylamine (TEMPOL-H), the maximal tolerated dose in female CH3 mice was 325 mg/kg. Tempol-H provided protection against the lethality of whole-body radiation in C3H mice at 30 d with a dose modification factor of 1.3, which is similar to the results obtained with TEMPOL. However, TEMPOL-H produced little effect on blood pressure or pulse compared with TEMPOL. Thus, TEMPOL-H is also a systemic in vivo radioprotector of C3H mice associated with less hemodynamic toxicity than TEMPOL [155]. TEMPOL supplementation to old Fischer 344 rats decreased plasma glucose, insulin, and triglycerides, unlike vehicle-supplemented old rats, and alleviated insulin resistance [156]. The amelioration of cardiac hypertrophy induced by hypoxic conditions [138] may suggest a possibility of using nitroxides in the prophylaxis of adverse effects of hypoxic conditions, e.g., in high mountain climbers.

Many reports document the protective effects of nitroxides in the ischemia-reperfusion injury of various organs.

Inflammation generates a multitude of reactive nitrogen and oxygen species, apart from cytokines, chemokines, and growth factors. Nitroxides show anti-inflammatory properties by scavenging these reactive species and many studies demonstrated their anti-inflammatory action. Apart from scavenging ROS and RNS, nitroxides also modulate directly the NF-κB factor considered to be a master regulator of inflammation, as shown for TEMPOL [157]. In prostate cancer progression, TEMPOL reduced inflammation in preclinical models, downregulated the initial inflammatory signaling via Toll-like receptors, and upregulated iκB-αand iκB-β levels, leading to a decrease in NF-κB, TNF-α, and other inflammatory markers [153]. TEMPOL was also reported to inhibit myeloperoxidase, which plays a fundamental role in oxidant production by neutrophils [158].

Neurodegenerative diseases are accompanied by oxidative stress, which has been suggested to contribute to their development [159,160]. Various reports document the potential protective effects of nitroxides in cellular and animal models of neurodegenerative diseases. TEMPOL inhibited lipopolysaccharide-induced β-amyloid (Aβ) formation in mouse hippocampal HT22 cells and mouse hippocampus [161]. In a mouse model of Alzheimer’s disease, a pyrrolyl α-nitronyl nitroxide (Figure 1, #19) attenuated brain Aβ deposition, and tau phosphorylation, decreased astrocyte activation and improved spatial learning and memory, being more effective than TEMPO [89]. TEMPOL was reported to prevent vascular response impairment and normalize astrocyte Ca^2+^ levels in APP mice, a mouse model of Alzheimer’s disease [162].

Nitroxide-containing nanoparticles administered to mice attenuated cognitive deficits of both spatial and non-spatial memories, reduced oxidative stress, and decreased Aβ(1–40), Aβ(1–42), and gamma (γ)-secretase levels, and Aβ plaque (see below).

The intraperitoneal administration of TEMPOL induced hypotension in C3H mice as well. This vasodilatory effect could contribute to its radioprotective effect in vivo since it can cause relative bone marrow and tissue hypoxia, resulting in decreased sensitivity of the bone marrow to ionizing radiation [116]. Interestingly, vaporized TEMPO was able to protect mice against ischemic damage. Other less volatile piperidine nitroxides were not effective [75].

Apparently, one of the most important results concerning the effects of nitroxides at the organismal level concerns the prolongation of the lifespan of tumor-prone mice [84,104,105]. The increased latency to tumorigenesis in Atm-deficient mice was associated with reduced oxidative stress and damage in cancer-prone tissues, suggesting that the chemopreventive effects of TEMPOL resulted from the reduction in oxidative stress and damage. The increased latency to tumorigenesis was greater in Atm2/2 (100%) than in p532/2 (25%). However, p53-deficient mice, which do not display an oxidative stress phenotype but are cancer-prone, and TEMPOL treatment of p532/2 mice did not have any effect on oxidative stress and damage. TEMPOL directly affected p53, increasing p53 phosphorylation at serine 18. TEMPOL also induced p21 protein expression. It was suggested that the chemopreventive effects of TEMPOL are not only due to modulation of oxidative stress and damage but, at least in part, to activation of the p53 pathway and modulation of redox-mediated signaling [84].

## 7. Clinical Trials of Nitroxides

By altering the redox status of tissues, nitroxides have the ability to interact with and alter many metabolic processes. Positive results of animal experiments justified clinical trials on nitroxides [163] that have been conducted.

TEMPOL was found to acutely and rapidly (within 30 min) improve the thermal hyperemia response in young adult smokers, returning the response back to that typically observed in healthy non-smokers and effectively reversing their impaired endothelial function observed. This effect was found to be entirely nitric oxide-dependent due to the decomposition of superoxide and protection of nitric oxide from inactivation by superperoxide. The various TEMPOL-based nitroxide drug candidates, which can be best used to improve cutaneous microvascular function or reduce the cardiovascular burden of cigarette smoking in humans, remain to be steadily investigated [164].

TEMPOL-based piperidine nitroxides may have similar effects on the aging microvasculature. TEMPOL was reported to attenuate the reduction in thermal hyperemia caused by infusion of angiotensin-II in young adults. Angiotensin-II is elevated with advanced age as well as in many disease states and induces oxidative stress by activating NADPH oxidase and xanthine oxidase. Hence, infusion of angiotensin-II mimics an aging state [165]. TEMPOL and apocynin, an inhibitor of NADPH oxidase, ameliorated the impaired thermal hyperemia observed in chronic kidney disease, another disease state characterized by high oxidative stress [166].

OT-551 (1-hydroxy-4-cyclopropanecarbonyloxy-2,2,6,6-tetramethylpiperidine hydrochloride, TEMPOL-H prodrug; Figure 1 #22) is a small molecule with antioxidant and anti-inflammatory effects. The protective efficacy of OT-551 and its metabolite TEMPOL-H against AMD was tested. A topical daily eye drop for AMD patients was capable of significant preservation of both standard luminance visual acuity and low luminance visual acuity, which is a measure of impaired night and reduced light vision, as evidenced in human Phase II clinical trials for dry AMD. The initial National Eye Institute (NEI), open-label single center was an NEI Intramural Research Program-sponsored Phase 2 clinical trial for OT-551 in dry AMD, which achieved statistical significance for the primary endpoint of preserving visual acuity [167].

The clinical application of TEMPOL was also evaluated in a pilot study at the University of Pennsylvania in which eleven patients with metastatic cancer to the brain were treated with topical TEMPOL. The nitroxide (70 mg/mL in water, ethanol and hydroxylpropyl cellulose) was applied topically to the scalp 15 min before and washed off immediately after the completion of each of 10 fractions of whole brain radiation. These results demonstrated that topical application of TEMPOL to the scalp before whole brain radiation is safe and well tolerated. The evidence of protection against radiation-induced alopecia was observed. A phase II study that uses a gel formulation to increase the exposure of the scalp to TEMPOL has been initiated [168]. A cyclosporine A—TEMPOL topical gel was proposed to be a highly promising platform for treating alopecia [169].

TEMPOL applied in a topical gel was also found to be tolerable when used to manage dermatotoxicity in patients with localized anal cancer undergoing chemoradiation. A total of 5 patients received topical TEMPOL. Adverse events attributed to TEMPOL included asymptomatic grade 1 hypoglycemia and grade 1–2 diarrhea. Dermatitis within untreated, radiated skin was not more severe than dermatitis in MTS-01-treated, unirradiated skin. Examples of clinical application of nitroxides are shown in Table 5.

## 8. Nitroxide-Containing Redox Nanoparticles

Low-molecular-weight nitroxides may pose various problems, including the limited life in the body due to reduction, metabolic transformations their rapid clearance by the kidney. For these reasons, they sometimes cannot fully exert their potent antioxidant capacity in vivo. To overcome these problems, redox polymers with covalently conjugated nitroxides were designed. If their size is in the nanometer range, they are referred to as nanoparticles. Several types of nanoparticles containing nitroxides have been synthesized.

Pluronic silica nanoparticles having nitroxide moieties covalently bound to the silica core protected by poly(ethylene glycol) chains (PluS–NO) via a TEMPO–CONH–R link and coumarin dyes embedded in the silica core reacted with peroxyl radicals with a rate constant of (1.5 ± 0.4) × 10^5^ M^−1^ s^−1^. As each PluS−NO particle bears an average of 30 nitroxide units, this yields an overall ≈60-fold larger inhibition of the PluS−NO nanoantioxidant compared to the molecular analog [11].

CdSe quantum dots functionalized with TEMPAMINE were proposed to act as free radical sensors due to efficient fluorescence quenching by TEMPAMINE and restoration of fluorescence upon reaction of TEMPAMINE with free radicals to form non-paramagnetic species [171].

The synthesis of two kinds of amphiphilic block copolymers, which can self-assemble into micelles with nitroxyl radicals-containing segments in the core, was reported [172]. Their diameter was <100 nm.

The synthesis of rotaxane-branched radical dendrimers Gn-TEMPO (generation n = 1–3) with up to 24 TEMPO radicals as termini [173], mannose-TEMPO functionalized G4- polyamidoamine (PAMAM) dendrimers [174] and 3Gc0T zero generation dendrimer with a cyclotriphosphazene core functionalized with nitroxyl radicals [175] was reported. PAMAM dendrimers of G1.0 and 2.0 with over 28% TEMPO loading were synthesized and used to oxidize cellulose in water [176]. TEMPO-terminated polyurethane dendrimers (G0–G4) showed better ABTS^●^ scavenging and hydrogen peroxide-scavenging activity than TEMPOL [177]. However, no cellular effects of such dendrimers, as well as of other nanoparticles mentioned previously, have been reported so far.

Synthesis of TEMPO-coated gold nanoparticles (average diameter of 2.5 nm) was reported [178]. Gold nanoparticles (average size 40 nm) conjugated with TEMPO were effectively taken up by human mesenchymal stem cells, did not significantly impair the cell viability, reduced the ROS level in H_2_O_2_-treated cells, and promoted the osteogenic differentiation of these cells [179].

Fluorescently trackable liquid crystal nanoparticles synthesized from a nematic diacrylate liquid crystalline cross-linker, a derivative of the chromophore perylene and a polymerizable monoacrylate amphiphile with a carboxylate headgroup that caps the nanoparticle [180] were taken up by HeLa cells showing limited effect on their viability and reduced intracellular ROS level [181]. pH-sensitive nanoparticles loaded with curcumin protected curcumin against degradation and allowed for the delivery of minimally degraded curcumin to target regions [182].

Nano-sized sterically stabilized liposomes loaded with TEMPAMINE [183] proved to be efficient in inhibiting autoimmune encephalomyelitis in mice, as well as adjuvant-induced arthritis in rats [184,185,186] and exerted an anticancer effect [169].

TEMPAMINE conjugated to poly[oligo(ethylene glycol)methyl ether acrylate] (POEGA) or poly(2-hydroxyethyl acrylate) (PHEA) yielded nanoparticles of molecular weight of 4.5–33.4 kDa and average diameter 31 and 27 nm, respectively. They showed good biocompatibility, not compromising the viability of MRC-5 fibroblasts, not affecting erythrocyte osmotic fragility, and not inducing hemolysis [187].

The group of Nagasaki synthesized redox nanoparticles (RNPs), which are self-assembling polymeric micelles with a diameter of about 40 nm using poly(ethyleneglycol)-b-poly[4-(2,2,6,6-tetramethylpiperidine-1-oxyl)oxymethylstyrene](PEG-b-PMOT) diblock copolymer. In aqueous media, the poly(ethylene glycol) (PEG) segments form an outer layer shielding the hydrophobic core containing the nitroxide residues [188]. By coupling TEMPOL or TEMPAMINE residues, they produced two types of RNPs: RNP^O^ with conjugated TEMPOL residues and RNP^N^ with conjugated TEMPAMINE residues. The latter are pH-sensitive since, at below 7.0, the amide bond becomes ionized, which results in the disintegration of micelles and an improvement in ROS scavenging activity. Thus, RNPNs can exert their action locally, at inflammation sites, or in the acidic tumor environment [189].

A range of beneficial effects of RNP was reported (Table 6 and Table 7), including anticancer effects and protection against pathological conditions involving oxidative stress, including ischemia-reperfusion injury [190,191,192,193,194,195,196]. While low-molecular weight nitroxides show dose-related antihypertensive action accompanied by reflex tachycardia, increased skin temperature, and seizures [120], both RNP^N^ and RNP^O^ did not induce any decrease in the arterial blood pressure.

## 9. Safety and Adverse Effects of Nitroxides and Nitroxide-Containing Nanoparticles

The toxicity of nitroxides is generally low. The IC_50_ values reported for HACaT cells were 2.66 mM for TEMPO, 9.5 mM for TEMPAMINE, and 11.4 mM for TEMPOL [49]. However, lower IC_50_ values were reported for other cell types. The IC_50_ values of TEMPOL for normal and malignant lung cells were 1–2 mM [100] and for *Leishmania* promastigotes to ca. 0.66 mM [100]. The higher sensitivity of malignant cells or parasites may be advantageous, provided that normal cells of the host are more resistant. Nitroxides can be given to animals in drinking water or food. TEMPOL administered in food (10 mg/g food) chronically was well tolerated and prolonged the life span of tumor-prone mice [84,104,105]. TEMPOL, injected intraperitoneally, was tolerated by female C3H mice up to a dose of 275 mg/kg. Above this dose, TEMPOL was lethal to varying degrees [59]. In acute animal experiments, nitroxide doses of 300 mg/kg or even 400 mg/kg [117] were applied. Nitroxides have a short time of circulation in blood. TEMPOL showed a fast decay in blood, and the EPR signal could not be detected approximately 5 min after injection, due to uptake by tissues and reduction. Metabolism of nitroxides takes place mainly in the liver, where TEMPO can be transformed into a five-membered ring in liver microsomes; the metabolites are excreted mainly into the bile [206].

In contrast to low-molecular-weight nitroxides, nitroxides bound to a macromolecular scaffold have much longer circulation time. The half-life of the RNP^N^ was 60 times longer (15 min) than that of TEMPOL. RNP^O^s show much longer circulation: the half-life of the RNP^O^ was 600 min, i.e., 2400 times longer than that of TEMPOL [190,191]. This effect seems to be partly due to the interaction of RNP with blood plasma proteins [193]. There are obvious conditions that must be fulfilled by an artificial biomaterial to be applied in vivo, including biocompatibility, adequate stability, and safety [207]. The redox PEG-b-PMNT nanoparticles containing nitroxides were reported to be even less cytotoxic than free nitroxides, not affecting cell viability up to a concentration of 8 mmol nitroxide residues/L and no mice toxicity at a dose of 300 mg/kg [190]. They can be given to animals in the food or drinking water [190,191]. The low toxicity of these nanoparticles is attributed to the fact that the outer PEG layer constitutes a stealth shield around the nitroxide moieties in the RNP core. However, the toxicity and safety of nanoparticles depend on their composition as they or cells containing them are ultimately subject to phagocytosis, their components are degraded, and metals, if present, are released.

## 10. Conclusions and Perspectives

The field of nitroxide research is continuously expanding, from chemical and physical studies of their properties via cellular effects to animal experiments and clinical applications. Their antioxidant properties, allowing them to react with free radicals and other ROS, in particular decompose superoxide radicals and prevent the Fenton reactions, justify their broad use in biology and medicine to alleviate oxidative stress. The decomposition of superoxide by piperidine nitroxides may be useful for the protection of blood nitric oxide and lower blood pressure. These compounds are used as NMR contrasting agents in NMR imaging and for EPR imaging (the latter fields of application were not the subject of this review). They are elements of nanoparticles prolonging their lifetime in vivo, allowing for the targeting of inflammation and tumor areas. There are reasons to expect that nitroxide-containing redox nanoparticles can be next-generation antioxidants. Adverse effects of nitroxides are generally not serious, and their cytotoxic effects may be useful for eliminating malignant cells and parasites. Presently, Pubmed responds to the item “nitroxides”, showing over 6300 publications; this number is expected to grow rapidly with the identification of new possibilities of experimental and therapeutic applications of these compounds.

## Figures and Tables

**Table 1:** Reaction rate constants of several pyrimidine nitroxides with peroxyl radicals. After [11] and others.

Substituent at C4	Reaction Rate Constant [M^−1^ s^−1^]	Standard Redox Potential with Respect to NHE [V]
-H	(5.1 ± 1.5) × 10^6^	0.722 [11]; 0.577 [13]; 0.833 [12]
-OH	(1.1 ± 0.5) × 10^6^	0.810 [11]; 0.603 [13]; 0.898 [12]
-NH_2_	(5.4 ± 1.5) × 10^5^	0.826 [11]; 0.789 [13]
-COOH	(3.7 ± 1.0) × 10^5^	0.82 [11]
-NHCOCH_3_	(1.1 ± 0.4) × 10^5^	0.88
-CONHBut	(1.9 ± 0.5) × 10^5^	
=O	(5.6 ± 1.2) × 10^4^	0.913
Trolox	(2.6 ± 0.7) × 10^5^	

**Table 2:** Protective effects of nitroxides in cellular systems or organ culture.

Cellular Model	Nitroxide, Concentration	Effect	Reference
CHO AS52 cells treated with H_2_O_2_ and hypoxanthine/xanthine oxidase	TEMPOL, 10 mM	Protection against mutagenic effects; no mutagenicity of TEMPOL alone	[69]
Cardiac ventricular cells from 1-day-old rats treated with hypoxanthine + xanthine oxidase	TEMPOL, 0.2 mM or hydroxylamine of TEMPOL 0.2 mM	Protection against contractability loss and LDH release by both nitroxide and hydroxylamine	[76]
Rat tracheal epithelial cells, exposed to NO donor *S*-nitrosoglutathione monoethyl ester, Angeli’s salt, SIN-1, and peroxynitrite	TEMPO, 14 and 15, 5 μM	Protection against DNA damage (tail moment in the comet assay)	[77]
B14 fibroblasts treated with 0.5 μM doxorubicin or 7 μM H_2_O_2_	TEMPO, TEMPOL, TEMPAMINE, 4-acetamido-TEMPO (Figure 1, #1–3 and 6)	0.05–3 mM nitroxides; protection against doxorubicin toxicity; 0.1–0.5 mM TEMPAMINE and 4-acetamido-TEMPO, protection against H_2_O_2_ toxicity (cell survival)	[78]
Human epithelial cell line A549 exposed to 0.1 ppm of O_3_ for 30 min	Cells pretreated with 100 μM TEMPO	Reduction in IL-8 production	[79]
A459 cells treated with 100 μM ferrous ammonium citrate for 24 h	0.25 μM 2-(2,4-dimethoxyphenyl) 4,4,5,5-tetra-methylimidazoline-1-oxyl-3-oxide) (Figure 1, #20) present during the treatment	Increase in viability; decrease in lipid peroxidation; attenuation of the decrease in GSH level; decrease in apoptotic rate	[80]
Rat pleural macrophages treated with 1 mM H_2_O_2_ for 4 h	TEMPOL 0.03–3 mM administered 15 min before H_2_O_2_	Attenuation of decrease in MTT reduction	[81]
HEK-2 cells treated with 80 μM t-BHP, or hypoxia (≤1% O_2_ for 24 h)/reoxygenation for 2 h)	Mito-TEMPO, 25 nM	Decreased ROS level; attenuation of the decrease in the level of mitochondrial transcription factor A and mtDNA copy number	[56]
Rat lenses treated with 1 mM H_2_O_2_ and incubated for 24 h	4 mM TEMPOL hydroxylamine	Prevention of opacification	[82]
Human dermal fibroblasts subjected to 340–400 nm UV	TEMPOL, 0.03–8 mM	Increase in cell survival; decrease in TBARS level; inhibition of MMP-1 and MMP-3 expression	[57]
Human RPE-19 cells with accumulated lipofuscin fluorophore A2E	OT-674 (hydroxylamine of TEMPOL), 0.01–10 mM	Protection against the death of blue–light-exposed A2E-laden cells	[73]
Chinese hamster cells, X-irradiated under aerobic conditions	TEMPOL, 5, 10, 50, and 100 mM, added 10 min prior to irradiation	Protection of cell viability; protection factor of 1.25, 1.30, 2.1, and 2.5, respectively	[58]
Chinese hamster V79 cells, irradiated with 4 MV photons	Nitroxides, 10 mM, given 10 min before irradiation	Protection of cell viability, protection factor at 10% survival: 2.4 for 3-aminomethyl-PROXYL, 2.3 for TEMPAMINE, 1.6 for 3-cyano-PROXYL, 1.5 for 3-carbamylo-PROXYL, 1.3 for TEMPOL, and 1.2 for 4-oxo-TEMPO	[83]
Thymocytes from 1-month-old p53^+/+^ and p^53−/−^ mice, 2.5 Gy of IR	TEMPOL, 1 mM, added 30 min before irradiation	Increase in p53 phosphorylation at Ser18 and p21 expression	[84]
Human fibrosarcoma HT1080 cells, ferroptosis induced by RSL3, and oxytosis induced by 5 mM glutamate	TEMPO 1, 2.5, 10, and 100 mM in a next dish, 37 °C, 5 h/8 h in the case of oxytosis	Concentration-dependent inhibition of ferroptosis by volatile TEMPO	[75]
Human neuroblastoma SH-SY5Y cells, differentiated, treated with 30 μM 6-hydroxydopamine (6-OHDA) (a cellular model of Parkinson’s disease)	TEMPOL, 30 μM	Attenuation of decrease in viability; decrease in necrosis; increase in mitochondrial superoxide production; increase in TBARS level; HO-1 expression; and NFκB-p65 activation	[60]
Human neuroblastoma SH-SY5Y cells, not differentiated, treated with 30 μM 6-OHDA	TEMPO, 100 and 150 μM; TEMPAMINE, 75–150 μM	Increase in cell survival; increase in GSH level; attenuation of decrease in mitochondrial potential	[62]
SH-SY5Y cells transfected with the tau protein (a model of chronic cellular oxidative stress)	TEMPO. TEMPOL and TEMPAMINE 1–10 μM	Decrease in the ROS level; increase in mitochondrial potential	[85]
Dopaminergic MN9D cells, differentiated, treated with 100 μM 6-OHDA for 20 min	TEMPOL, 0.15 μM, added 1 h before 6-OHDA	Protection of cell viability; activation and nuclear translocation of NF-κB	[61]
Bovine aortic endothelial and smooth muscle cells exposed to 23 mM glucose	5 mM TEMPOL	Increase in GLUT1 and GLUT4 expression and in glucose uptake	[63]
Immortalized mouse podocytes, treated with high glucose (30 mM) for 48 h	TEMPO, 100 nM	Inhibition of triglyceride and cholesterol accumulation	[64]
Inflammatory neutrophils isolated from Swiss male mice administered i.p. with 12% sodium caseinate	TEMPOL 5–120 μM TEMPOL 120 μM	Inhibition of respiratory burst, IC_50_ = 45 μM; inhibition of protein kinase activities, inhibition of fungicidal activity	[86]
Buffalo bull spermatozoa	Mito-TEMPO 50 μM added to semen extender	Improvement in post-thaw semen quality; reduction in ROS and TBARS levels	[67]
Ram’s semen cryopreserved and thawed	Mito-TEMPO added to the cryopreservation medium, 5 and 50 μM	Improvement in sperm motility, membrane functionality, and mitochondrial activity; attenuation of apoptotic changes; increase in ROS and TBARS levels	[65]
Ram’s semen in the cooling medium stored at 5 °C for 24 and 48 h	Mito-TEMPO added to the medium, 5 and 50 μM	Improved sperm viability, motility, and mitochondrial membrane potential; decreased TBARS level, elevated pregnancy, parturition, and lambing rates	[66]
Bovine oocytes in the maturation medium	1 μM mito-TEMPO in the medium	Increase in the proportion of developing oocytes and expression of Bcl2 and GSH level; decrease in the ROS level and expression of Bax; better effect of mito-TEMPO than TEMPO	[68]
Baker’s yeast *Saccharomyces cerevisiae*	TEMPO, 3 mM	Reduction in the number of double-strand DNA breaks; increase in metabolic rate; change in the pattern of gene expression; slowing down the aging of postmitotic cells; protection against H_2_O_2_ toxicity	[87]
*Candida albicans*	TEMPOL, 0.344 mg/mL	Upregulation of genes involved in iron homeostasis, mitochondrial stress, steroid synthesis, and amino acid metabolism; IC_90_: 0.5–0.68 mg/mL	[88]
Biofilms of *Staphylococcus aureus*	Nitroxides conjugated to antibiotics, e.g., fluoroquinolone-TEMPO or coadministration of 4-carboxy-TEMPO with ciprofloxacin	Penetration of biofilms and into the cells; dispersal of biofilms; no toxicity to human cells	[24]
Mouse primary neurons	0.1–10 μM L-NNNBP	Protection from the toxicity of 5 Aβ1–42 and Aβ1–42-induced caspase activation and apoptosis, protein nitration, and depolarization of mitochondria	[89]
Cytoplasmic hybrids (cybrids) of SH-SY5Y cells containing mitochondria from platelets of patients with mild cognitive impairment and cortical neurons from tau mice	Mito-TEMPO (concentration not indicated)	Protection of mitochondrial respiratory function; suppression of tau oligomer accumulation	[90]

**Table 3:** Pro-oxidant and adverse effects of nitroxides in cellular systems.

Cellular Model	Nitroxide, Concentration	Effect	Reference
HaCaT cells	Nitroxides, 24 h treatment	Cytotoxicity, IC_50_ values: TEMPO, 2.66 mM; TEMPOL, 11.4 mM; TEMPAMINE, 9.5 mM	[49]
Human erythrocytes, hematocrit 20%, 3-h incubation at 37 °C	TEMPO, 0.5 mM	Decrease in GSH level down to 30% after 3 h, increase in GSH export to the extracellular medium, increase in methemoglobin content	[92]
Human erythrocytes, hematocrit 10%, 1-h incubation at room temperature	TEMPO 0.2–2 mM	Human erythrocytes, hematocrit 10%, 1 h incubation at room temp.	[93]
Rat glioma C6 cells	TEMPOL, 1 mM	60 and 67% apoptosis after 24 and 72 h, respectively	[94]
Human prostate carcinoma PC-3, LNCaP and DU-145 cells	TEMPO 0.25–5 mM	Induction of apoptosis; increase in activities of caspases 3 and 9; chromatin fragmentation; viability loss	[95]
MCF-7. HL-60, HepG2 cells	5 mM TEMPO	Increase in the intracellular level of H_2_O_2_	[95]
Bovine aortic endothelial and smooth muscle cells, 5 and 23 mM glucose	0.2–5 mM TEMPOL 5 mM TEMPOL	Increase in ROS level; increase in protein carbonylation	[63]
Microvascular cells derived from bovine adrenals, X-irradiated (8 Gy)	0.5 and 2 mM TEMPOL, 10 min before and 1 h after irradiation	Partial prevention of cell mobility loss in a wound healing assay	[96]
B14 fibroblasts	5-DS 0.5–500 μM, Methyl-12-DS, and 16-DS, 0.5 μM–2 mM, 24-h incubation	Reduction in viability	[97]
Vero E6 cells	TEMPOL, 0.2 and 0.5 mM	Loss of the Fe-S clusters of nsp12; no effect on the activities of several mitochondrial Fe-S enzymes, including the respiratory complexes, mitochondrial aconitase, and cytosolic dihydropyrimidine dehydrogenase	[72]
Immortalized human keratinocytes, HaCaT	TEMPOL, TEMPOL-H, TEMPAMINE 5 mM; TEMPO and TEMPO+, 16 μM TEMPOL, TEMPOL-H, TEMPAMINE 5 mM; TEMPO, 1 mM; TEMPO^+^, 16 μM TEMPOL, TEMPOL-H, TEMPAMINE 5 mM; TEMPO, 1 mM; TEMPO^+^, 4 μM	Increase in the level of ROS. Activation of Nrf2; (Small) protection against UV (30 mJ/cm^2^)	[91]
PC3 human prostate cancer cells and HT29 human colon cancer cells	TEMPAMINE conjugates of betulinic, fusidic, and cholic acids	Significant cytotoxicity, 6.0 and 7.4 μM, respectively, for the fusidic acid conjugate, a mitochondria-targeted fusidic acid derivative constructed	[98]
Human squamous lung carcinoma Calu-6 cells, non-squamous lung carcinoma A549 cells, and normal lung WI-38 VA-13 subclone 2RA cells	TEMPOL, 48 h	IC_50_ of 1–2 mM, no difference between carcinoma and normal cells Increase in ROS, GSH depletion (2 mM TEMPOL)	[99]
Baker’s yeast *Saccharomyces cerevisiae*	TEMPO	Growth inhibition of Δ*sod1* strain (from 0.1 mM TEMPO and Δ*sod2* strain (from 1 mM TEMPO; wild type yeast from 3 mM TEMPO	[87]
Promastigotes of *Leishmania braziliensis* in macrophages	TEMPOL	Killing of promastigotes with IC_50_ of 0.66 mM	[100]

**Table 4:** Effect of nitroxides in experimental animals.

Animal Model	Nitroxide, Dose	Effect	Reference
C3H mice	TEMPOL administered in chow, 10 mg/g food (ca. 58 mM)	Weight reduction (28.2 ± 0.8 g vs. 41.9 ± 0.6 g over 30–75 weeks) Increase in mean life span (123 vs. 92.6 weeks) Longer persistence of activity and coat color Decreased tumor incidence (10% vs. 40%) Elevated levels of UCP-2 and HSP70 in the skeletal muscle	[104]
Atm^−/−^ mice, in 129SvEv background, a mouse model of ataxia telangiectasia, displaying accelerated oxidative damage and stress	TEMPOL administered in chow, 10 mg/g food	No decrease in food intake, metabolic rate, or physical activity, but reduced body mass, decreased ROS, and increased mitochondrial potential in thymocytes, reduced proliferation of thymocytes and splenocytes, attenuation of increased HO-1 expression in the brain and thymus, and increased protein carbonyls	[105]
p53^−/−^ mice	TEMPOL administered in chow, 10 mg/g food	Increase in mean tumor-free survival (21.4 to 25 w), no reduction of oxidative stress	[84]
Sprague Dawley male rats administered intragastrically with 1 mL 96% ethanol, subcutaneously with indomethacin (30 mg/kg b.w.), or intragastrically with aspirin (0.1 g/kg b.w.)	TEMPOL 0.1 g/kg b.w. 5 min before induction of the damage	Reduction in mucosal damage and the level of leukotriene B_4_ in the mucosa	[106]
Male C57BL/6 mice, renal ischemia induced by bilateral clamping of the renal pedicles for 30 min	Mito-TEMPO, 25 μL of 5 μM solution injected into each kidney after reperfusion, and then 5 mg/kg each day, i.p. for 5 days	Improvement in renal functions decreased Bax expression	[56]
Hearts isolated from male Sprague Dawley rats, 10 min of ischemia, 5 min reperfusion	TEMPO, 0.4 and 1 mM in the perfusion fluid from 10 min before reperfusion	Protection against reperfusion injury and LDH release	[106]
Male Sprague Dawley rats, heart ischemia (5 min) and reperfusion (30 min)	TEMPOL 30 mg/kg and 100 mg/kg, 5 min before occlusion, 60 s before reperfusion, or 60 s after onset of reperfusion	Protection against ventricular tachycardia and ventricular fibrillation when administered before ischemia or before reperfusion	[107]
Dogs subjected to 20-min cardiac arrest	TEMPOL 300 mg/kg in saline flush	Improved cerebral performance, better neurologic scores	[108]
Male Wistar Albino rats, a 60 min occlusion of the superior mesenteric artery	TEMPOL 30 mg/kg in saline solution during the first 60 min of reperfusion	Attenuation of increase in myeloperoxidase activity, TBARS level, bacterial translocation, and decrease in GSH level	[109]
Male Wistar rats, subjected to 30-min liver ischemia and 2-h reperfusion	NNR (Figure 1, #21), 30 mg/kg 10 min before reperfusion and 1 h after the onset of reperfusion	Decrease in hepatic TBARS level and serum level of ALAT and ASPAT	[110]
Male Wistar rats, two-kidney, one-clip (2K-1C; 8 w) hypertension	TEMPOL 18 mg/kg/day by gavage	Attenuation of increase in systolic blood pressure, reduction in endothelium-dependent vasorelaxation and MMP-2 activity, vascular remodeling, ROS, and TBARS levels	[111]
Wistar rats subjected to bilateral renal occlusion for 45 min	TEMPOL 30 mg/kg	Attenuation of increase in total severity score plasma urea and creatinine and LDH release	[112]
Male Mongolian gerbils, ischemia-reperfusion injury induced by 5-min bilateral occlusion of the common carotid arteries	TEMPOL, 30 mg/kg i.p., 30 min before and 1, 2, and 6 h after the onset of reperfusion	Increased survival, reduced hyperactivity, reduced nitrotyrosine staining, reduced loss of neurons from the pyramidal layer of the CA1 region	[81]
Male Sprague Dawley rats, ischemic acute renal failure induced by renal artery and vein were occlusion for 45 min	TEMPOL 100 mg/kg, i.v, 5 min before ischemia	Attenuated the ischemia/reperfusion-induced renal dysfunction	[113]
C.B-17/Icr-^+/+^Jcl mice, focal cerebral ischemia induced by electrocoagulation	0.1 g of cotton soaked in 5 mL of 0.03–1 mM TEMPO in mouse cage 15 min after infarction for 8 h	Reduction in ischemic damage	[75]
Female Sprague Dawley rats, acute retinal ischemia by elevation of intraocular pressure for 60 min	5,6-dicarboxy-1,1,3,3-tetraethyllisoindolin-2-yloxyl, 2 μL of 2.5 mM solution injected into eye 30 min before reperfusion, 2 i.p. injections (20 mg/kg) at the beginning of experiment and after 60 min reperfusion	Protection of the retina from I/R-induced damage, maintaining retinal function, and decrease in the number of “activated” microglia, particularly in the outer retina	[113]
Sprague Dawley rats exposed to 700 lux of white fluorescent light for 6 h	OT-551, 25, 50, or 100 mg/kg; TEMPOL-H (OT-674, 100 mg/kg	Reduction in RPE damage index, more significant for all concentrations of OT-551 than TEMPOL-H	[114]
Sprague Dawley rats exposed to 700 lux of white fluorescent light for 6 h	OT-551, 25, 50, or 100 mg/kg; TEMPOL-H (OT-674, 100 mg/kg TEMPOL-H (OT-674, 100 mg/kg	Reduction in increase in the levels of 4-HNE- and 4-HNE-protein adducts, increased electroretinogram b-wave amplitudes, and increased outer nuclear layer thickness, more significant for all concentrations of OT-551 than TEMPOL-H	[115]
Female C3H mice subjected to ^137^Cs gamma irradiation (dose rate: 1 Gy/min)	TEMPOL administered i.p. 5–10 min prior to irradiation	Radioprotective effect: increase in LD_50_ from 7.84 Gy to 9.97 Gy	[59]
Female C3H mice irradiated with 9 kGy of γ radiation (^137^Cs source)	TEMPOL 275 mg/kg, TEMPAMINE 250 mg/kg, 3-aminomethyl-PROXYL 225–275 mg/kg, 3-carbamoyl-PROXYL 300–500 mg/kg, 4-oxo-TEMPO 225 mg/kg, and 3-CTPO 400 mg/kg given i.p. 5–10 min before irradiation	Increase in survival, 3-AM > TEMPOL > 3-CTPO > 3-CP > TEMPAMINE > 4-oxo-TEMPO. Decrease in blood pressure over 60 min, smallest for 3-CP	[116]
Female C3H mice with RIF-1 tumor	Whole-body irradiation, 10–60 Gy with 9 Me electrons TEMPOL i.p. injection (275 mg/kg, 10 min prior to irradiation	No protection of tumor cells, radioprotection of bone marrow cells, probably due to greater reduction to hydroxylamine in cancer cells	[117]
Female C3H mice subjected to localized X irradiation, 5 × 6 Gy to head Female C3H/Hen mice with propagated squamous cell carcinoma, X-irradiated 5 × 3 Gy or with HT-29 adenocarcinoma, X-irradiated 5 × 2 Gy	TEMPOL i.p. injection (275 mg/kg) 10 min before irradiation + 50 μL TEMPOL gel to oral cavity TEMPOL i.p. injection (275 mg/kg) 10 min before irradiation + 50 μL TEMPOL gel to oral cavityo oral cavity	Salivary gland radioprotection No tumor radioprotection; 2× faster reduction to hydroxylamine in the cancer	[118]
Female C3H mice, head irradiated with X-rays or carbon-ion beam	TEMPOL 150 mM injected i.v.	Changes in redox status of the brain, reduction followed by reoxidation	[119]
Miniature pigs of both sexes (30–80 kg)	TEMPOL 25–35 mg/kg and 3-CP (10, Figure 1) 30–300 mg/kg given i.v.	Decrease in arterial blood pressure (maximal after 5–10 min) accompanied by increased heart rate. No hypotensive effect of 3-CP	[120]
Male Sprague Dawley rats, experimental hypertension induced by deoxycorticosterone acetate	TEMPOL, 15 mg/kg, i.p., 21 days	Alleviation of hypertension, improvement in acetylcholine-induced EDHF-mediated vasodilation	[121]
Obese Zucker rats	1 mM TEMPOL in drinking water for 15 d	Decreased body mass and the levels of insulin, triglycerides, and TBARS, improvement in insulin sensitivity	[122]
Zucker rats fed a high-fat diet for 10 w	TEMPOL, 1 mM in the drinking water for 10 w	Decrease in the levels of blood glucose, triglycerides, cholesterol, VLDL, CRP, insulin, and urinary albumin, increase in blood HDL, attenuation of the expression of genes coding for desmin, TNF-α, NFκB, and NOX-1	[123]
SOD1^−/+^ C57BL/6 mice, streptozotocin-induced diabetes	TEMPOL 80 mg/kg/d for 35 days	Suppression of albuminuria increases in glomerular transforming growth factor β, collagen α1(IV), nitrotyrosine, and glomerular superoxide	[124]
Female C3H/Hen^−^ TacMT mice fed high-fat diet	TEMPOL in the diet (10 g/kg)	Restriction of body mass gain and lipid accumulation, alteration of gut microbiome, downregulation of fatty acid synthesis genes, and upregulation of fatty acid oxidation genes	[125]
ApoE^−/−^ mice, fed standard and high-fat diet (HFD)	TEMPOL, 10 mg/g food, up to 90 d	Reduction in body mass gain in mice fed standard diet but, especially, HFD, decrease in plasma triglycerides and cholesterol and inflammatory markers.	[126]
Male Sprague Dawley rats, experimental hypertension induced by 28-d treatment with deoxycorticosterone	TEMPOL 1 mM administered in drinking water during the experiment	Amelioration of hypertension (142 ± 5 vs. 199 ± 3 mm Hg)	[127]
Sprague Dawley rats intraperitoneally injected with LPS to induce hypertension	TEMPOL 1 mM in drinking water	Prevention of hypertension in the first-generation offspring and the transgenerational inheritance of hypertension	[128]
C57BL/6J mice, 20–25 g, fed high fructose (8 weeks), subjected to transverse aortic constriction	TEMPOL, 0.1% in feed (ca 150 mg/kg/day), 8 weeks	Attenuation of cardiac hypertrophy, decrease in LV area; decrease in TBARS and 4-hydroxyalkenals	[129]
Male Sprague Dawley rats carrageenan-induced pleurisy	TEMPOL, 10, 30, and 100 mg kg^−1^ given i.p. 15 min before carrageenan	Dose-dependent attenuation of lung injury histology, increase in tissue myeloperoxidase and TBARS, decrease in nitrotyrosine content and peroxynitrite formation	[130]
Male Sprague Dawley rats, dinitrobenzene sulfonic acid-induced colitis	TEMPOL i.p., 15 mg/kg daily for 7 d	Decrease in mortality, damage score, myeloperoxidase activity, and TBARS level in the colon	[131]
C57BL/6 mice, experimental colitis induced by 3% *w*/*v* dextran-sodium-sulfate (DSS) in drinking water over 9-days	4-Methoxy-TEMPO, 15 mg/kg, i.p., twice daily	Decreased clinical index, attenuation of body mass loss, crypt loss, mucin loss; decreased cellular infiltrate and serum content of lipid peroxidation products	[132]
Male Sprague Dawley rats, zymosan-induced generalized inflammation	TEMPOL (100 mg/kg i.p.) at 1 and 6 h after zymosan administration	Decreased mortality, toxicity score, myeloperoxidase activity, and TBARS level in lung, intestine, and liver	[133]
Male Lewis rats, collagen-induced arthritis	TEMPOL, 10 mg/kg/d, i.p., days 23–34	Decrease in % of arthritic rats, reduced hind paw swelling, histological damage score, radiograph score, and plasma level of TBARS	[134]
Male CF-1 mice subjected to controlled cortical focal traumatic brain injury	TEMPOL 300 mg/kg i.p. 15 min after the injury TEMPOL 300 mg/kg i.p. 15 min, 3, 6, 9, and 12 h)	Suppression of 3-nitrotyrosine formation in injured cortical tissue 1 h after injury Suppression α-spectrin degradation by 45% at 24 h	[135]
Male Sprague Dawley rats, periodontitis induced by ligation of the 1st molar for 8 d	TEMPOL, 10 mg/kg daily, i.p., for 8 days	Decreased neutrophil infiltration, tissue permeability, nitrotyrosine level, poly-(ADP-ribose)polymerase (PARP) activation	[136]
Lewis rats, glomerular immune injury induced by an antiglomerular basement membrane antibody or TNF	TEMPOL, 230 mg/kg, i.p.	Decrease in of urine protein and total isoprostane excretion	[137]
Male Wistar rats subjected to hypoxic, hypobaric conditions for 2 weeks	TEMPOL, 1 mM in the drinking water during the experiment	Prevention of increase in the right ventricular systolic pressure, amelioration of right ventricular hypertrophy	[138]
A/J mice, 6-OHDA administered to the striatum (a model of Parkinson’s disease)	TEMPOL, 200 mg/kg, given i.p. 60 min before the treatment	Reduced ptosis score, increased activity score, decreased fractional mortality	[61]
Zebrafish (*Danio rerio*) microinfected with *Mycobacterium marinum* (a model of tuberculosis)	4-Metoxy-TEMPO, 1 and 5 mM in the medium	Inhibition of production of mitochondrial ROS decreased infection-induced granuloma cell death, disruption of the NADH: NAD^+^ balance in *M. marinum*	[139]
Female prepuberal Sprague Dawley rats treated with dehydroepiandrosterone for 21 d	TEMPOL 30 mg/kg daily for 12 d	A significant reduction in intestinal oxidative stress in polycystic ovary syndrome rats without affecting the ovarian redox state. Changes in gut microbiota composition and serum metabolite profiles	[140]
Female C57BL/6 mice injected with 0.52 × 10^6^ cells of *Candida albicans*	TEMPOL, 1.6 mg/g of mouse/day	Partial protection, reduction in fungal burden in the kidneys of infected animals during infection onset, improvement in animal fitness	[88]
C57BL/6N mice	TEMPOL administered by gavage, 250 mg kg ^−1^ per day	Alteration in the gut microbiome, preferential reduction in *Lactobacillus* and its bile salt hydrolase activity, and anti-obesity effects	[141]
Female Sprague Dawley rats subjected to ventral root crush (VRC) at the lumbar intumescence	TEMPOL 250 mg/kg, 10 min and 24 h after injury and then every 48 h for 14 days	Preservation of proprioceptive glutamatergic inputs without exacerbating the rate of motoneuron degeneration	[142]
Female Wistar Kyoto (WKY) rats given dexamethasone (0.1 mg/kg per day) s.c. from gestational day 15 to 21)	TEMPOL, 1 mM in drinking water during pregnancy plus 100 μg/kg s.c., days 15–21	Attenuation of Dex-induced increases in blood pressure, adrenal mRNA, and protein levels of catecholamine biosynthetic enzymes in the offspring	[143]
Male Wistar rats administered bisphenol A, 10 mg/kg b.w., orally, once a week for 4 w	Mito-TEMPO 0.1 mg/kg b.w, i.p. twice a week	Normalization of sperm parameters and preserved histoarchitecture of the testis, inhibition of increase in mitochondrial ROS level and lipid peroxidation	[144]
Male Fisher 344 rats, 4-week simulated weightlessness, 0.75 or 1.5 Gy of cosmic radiation, 12–13-month recovery	Tissues (distal internal pudendal artery and corpus cavernosum) incubated with 5 μM mito-TEMPO for 30 min	Improvement in neurovascular erectile function	[145]
C57BL/6 mice, sepsis induced by i.p. injection of LPS, 5 mg/kg b.w.	LPS, 20 mg/kg, i.p., body,1h prior to LPS injection	Inhibition of inflammation, attenuation of LPS-induced liver injury, prevention of increase in serum TBARS level, attenuation of increase in mitochondrial ROS production	[146]
Male C57Bl/6 and Balb/c mice, asthma induced by chicken ovoalbumin or house dust mites	3-CP in chow (1% *w*/*w*) during the experiment	Decrease in the inflammatory cell count, pulmonary collagen, TGF-β and 3-nitrotyrosine, improved baseline pulmonary functions	[147]
C57Bl/6 mice, bleomycin-induced lung injury	3-CP in chow (1% *w*/*w*) during the experiment	Decrease in the inflammatory cell count, pulmonary collagen, fibrosis, TGF-β and 3-nitrotyrosine, improved baseline pulmonary functions and weight loss	[148]
C57BL/6 mice transfected with a vector coding for cardiomyocyte-specific mitochondrially targeted calpain 1	Daily i.p. injections of mito-TEMPO, 0.7 mg/kg/day, for 30 d	Inhibition of progression of progression of dilated heart failure, adverse myocardial remodeling, reduced mortality	[149]
HSV-1 virus in Vero (African green monkey kidney) cells	4-Substituted-1,2,3-^1^H-1,2,3-triazole linked TEMPOL derivatives	New derivatives are less cytotoxic than acyclovir, one more virucidal than acyclovir	[150]
C57BL/6 J mice subjected to 5/6 nephrectomy	Mito-TEMPO 1 mg/kg/d, i.p., 12 w	Improvement in impaired renal function and renal fibrosis, attenuation of kidney disease-induced muscle atrophy, suppression of inflammatory cytokines and ROS level, mitochondrial dysfunction, and endoplasmic reticulum stress in skeletal muscles	[151]
Male BALB/c mice, hepatocarcinogenesis induced by N-nitrosodiethylamine (i.p.)	Mito-TEMPO, 0.1 mg/kg, i.p., once a week until the end of the experiment	Increased animal survival (by 30%), decreased tumor incidence (by 25%), and tumor multiplicity (by 39%).	[152]
Athymic female nude mice injected with C6 glioma cells	TEMPOL, 0.25 g/mL (TPL B) receiving 0.375 g/mL via osmotic pump (0.5 μL/h) for 14 days plus 2 daily i.p. injections, 100 mg/kg), 5 days/week	Dose-dependent decrease in the xenograft growth	[94]
Male BALB/c nu/nu mice inoculated with LNCaP, DU-145, or PC-3 cells	TEMPO given intratumorally, once daily, 100 mg/kg per dose, Days 1–8, and then 200 mg/kg per dose, Days 9–23	Decreased tumor growth	[95]
Male TRAMP mice in the early and late stages of prostate cancer	TEMPOL 50 or 100 mg/kg diluted in water five times a week for 4 w	Decrease in NFB total protein and TNFα levels; decreased tumor progression	[153]
Male APP/PS1 double-transgenic mice (model for Alzheimer’s disease)	L-NNNBP, 1 mM in drinking water (55–100 mg/kg) for 1 m	Attenuated brain Aβ deposition and tau phosphorylation, decreased astrocyte activation, and improved spatial learning and memory	[89]
Male spontaneously hypertensive (SHR) rats	Pipridine and pyrrolidine nitroxides, i.v.	Piperidine but not pyrrolidine nitroxide dose-dependently decreased mean arterial pressure (by more than 40 mm Hg at 270 μmol/kg TEMPOL)	[154]

**Table 5:** Nitroxide effects in humans.

Examined Subjects	Nitroxide, Administration	Effect	Reference
Ten young smokers (19–26 y)	TEMPOL 10 μM, infused (2.0 μL/min) via microdialysis fibers to the ventral side of the forearm in the dermal layer of the skin for at least 75 min	Increase in the NO-dependent cutaneous vascular conductance plateau, indicative of enhanced NO availability	[164]
Eight healthy volunteers (24.5–29.5 y)	TEMPOL 10 μM, administered via a microdialysis catheter	Partial reversal of attenuation of the heat response (measure of local blood flow) caused by angiotensin-II, apparently due to superoxide scavenging	[165]
Twelve patients with metastatic cancer to the brain, irradiated with 3000 cGy delivered in fractions of 300 cGy/d	TEMPOL 70 mg/mL, administered topically to the scalp, 30–45 min each day	TEMPOL blood levels averaging from 0.4 to 0.7 mol/L; full scalp hair retention in 60% of the patients	[168]
Ten patients with chronic kidney disease, stages 3–4	TEMPOL 10 μM administered via intradermal microdialysis fibers	Augmentation of NO-dependent cutaneous vasodilation ascribed to superoxide scavenging	[166]
Ten patients with geographic atrophy, the advanced atrophic form of age-related macular degeneration (AMD)	Topical 0.45% OT-551 was administered in one randomly assigned eye three times daily for 2 y	The drug was well tolerated, with few adverse events; smaller decrease in the best corrected visual acuity	[167]
Five patients with anal carcinoma received X radiation, 42–45 Gy in 28 fractionsered as a single daily fraction	TEMPOL administered in a topical gel (70 mg/mL) 15–30 min prior to each fraction of radiation	Amelioration of dermatitis in the irradiated skin	[170]

**Table 6:** Cellular effects of nitroxide-containing nanoparticles.

Nanoparticle Composition	Structure	Model	Effect	Reference
Random dimethylacrylamide (DMA)- -TEMPO) copolymers	MW 1–19 kDa; Optimal composition: 40 mol % TEMPO/60 mol % DMA (MW 17.1 kDa)	ATDC5 chondrogenic cells	Protection from 1 mM SIN-1 induced cytotoxicity	[197]
TEMPAMINE conjugated to POEGA-pentafluorophenyl (PFP) functional nanostars	MW 94.1–130.8 kDa, diameter 10–20 nm	BJ-5ta fibroblasts and MCF-7 cells	No cytotoxicity up to 1 mg/mL; mitochondrial localization; decrease in cellular ROS level	[198]
TEMPAMINE conjugated to (POEGA) or poly(2-hydroxyethyl acrylate) (PHEA)	MW 4.5–33.4 kDa; average diameter 31 and 27 nm, respectively	MRC-5 fibroblasts Erythrocytes	No effect on viability up to 15 μM No hemolysis or change in osmotic fragility	[187]
RPN	Mean diameter of ca. 40 nm	SH-SY5Y cells treated with 6-OHDA	Protection of cell viability, attenuation of ROS increase, decrease in mitochondrial potential and ATP level	[62]
Magnetic silica nanoparticles, Fe3O4@SiO2 functionalized with (3-isocyanatopropyl) triethoxysilan or with fluorescein isothiocyanate, covered with dextran functionalized by TEMPOL, 1–25 μM in TEMPO	Size < 50 nm	Human microvascular endothelial cells, MDA-MB-231 breast cancer cells, yeast *S. cerevisiae*	Nanoparticles as a useful tool to study endocytosis	[199,200]
TEMPAMINE-dimethylacrylamide copolymer	MW of 1–19 kDa	ATDC5 chondrocyte-like cells treated with SIN-1	Polymer uptake, cytoprotection	[197]
Perylene (PY)-loaded liquid crystal NPs (PY-LCNPs) surface functionalized with poly (ethylene glycol) (PEG) and TEMPO	Mean diameter 145 nm; zeta potential of −1 mV; ca. 1880 TEMPO molecules per nanoparticle	HeLa cells treated with 0.5 mM H_2_O_2_ or t-BHP	Decreased level of intracellular ROS and lipid peroxidation	[181]

**Table 7:** Effects of nitroxide-containing nanoparticles in animal experiments.

Composition	Structure	Model	Effect	Reference
Poly(D,L-lactide-co-glycolide)-poly(ethylene glycol)-bis(amine)-folate conjugated with TEMPO or rapamycin and TEMPO	Mean size 153 nm, zeta potential of −28.2 mV	PKD (KspCre•Pkd2^flox^/^flox^) mice (animal model of bilateral renal cyst formation)	Increase in the efficacy, potency, and tolerability of rapamycin, increased survival rate, and improved kidney function	[201]
Nano sterically stabilized egg phosphatidylcholine-based liposomes loaded with TEMPAMINE	Mean size 74.3 nm; 7 mM TEMPAMINE, encapsulation >85%, drug to lipid ratio 0.16	SJL/J mice, acute encephalomyelitis (EAE) model	Limited therapeutic efficacy	[186]
PEGylated nano sterically stabilized egg phosphatidylcholine-based liposomes loaded with TEMPAMINE	Diameter about 80 nm	Female mice, acute EAE induced with proteolipid protein (PLP_139–151_). Chronic EAE induced with myelin oligodendrocyte glycoprotein (MOG)_35–55_ peptide	Improvement in clinical score in both models, decrease in mRNA of pro-inflammatory cytokines IFNγ and TNFα (chronic EAE)	[185]
RNP^O^	Diameter ca. 40 nm	Chicken embryo, 14 d old, treated with AAPH (and RNP), 4 mg/egg) effects studied after 72 h Treated with sodium hydrocortisone hemisuccinate sodium, 300 μg, RNP^O^ administered after 15 h, effects studied after 48 h	RNP^O^, 57 μg, protection against lethality RNP^O^, 300 μg, decreased TBARS in blood serum	[196]
RNP	Hydrodynamic diameter: 36.6 ± 0.1 nm, zeta potential: −16.2 ± 2.1	Zebrafish larvae exposed to hydrogen peroxide (1.5 and 4 mM) or AAPH (10 or 20 mM)	No discernible toxicity, significant improvement in survival under oxidative stress	[202]
RNP	Diameter ca. 40 nm	Male ICR mice, ischemia induced by left renal pedicle clamping for 50 min; RNP^N^ renal injection, 3 mg/kg or RNP^O^ 1.5 mg/kg, 5 min after reperfusion	Suppression of increase in superoxide, lipid peroxidation, and IL-6 in renal tissue and changes in blood pressure, the much stronger effect of RNP^N^	[203]
RNP	Diameter ca. 40 nm	hos: HRM2 hairless mice irradiated once a day for 5 d with UVB (302 nm); RNPN introduced into the skin by iontophoresis 3 times every 3 d before irradiation	Decreased UV-induced melanin production in the skin	[194]
RNP^O^ and RNP^N^	Diameter ca. 40 nm	Beagle dogs, cardiac ischemia (occlusion of the left anterior descending coronary artery for 90 min, followed by reperfusion (6 h); RNP^O^ (3 mg/kg) injected into a vein 5 min before reperfusion	Reduction in infarct size and myocardial apoptosis, increase in however coronary venous NOx	[204]
RNP^N^	Diameter ca. 40 nm	Male BALB/c mice inoculated with C26 murine colon cancer cells, X-irradiated; RNPN (200 mg TEMPO/kginjected s.c. 24 h before irradiation	Increased survival (10–30 Gy), prevention of kidney and liver damage. Intestine and bone marrow	[195]
RNP^N^	Diameter ca. 40 nm	Male BALB/c mice, male ICR mice injected (s.c.) with colon-26 colon adenocarcinoma cells; RNP^N^ (300 mg/kg) injected i.v.	Decreased generation of superoxide and TNFα in the tumor tissue, reduction in tumor volume growth, prevention of increase in plasma creatine kinase and MDA levels	[189]
RNP^N^	Diameter 30–40 nm	Tg2576 Alzheimer’s disease mice; RPPN, 5 mg/mL drinking water for 6 m	Improvement in memory and learning parameters, Attenuation of increase in MDA superoxide and 8-OHdG levels, decrease in GPX activity Aβ(1–40) levels in brain and plasma, and γ-secretase activity in brain	[205]
RNP^N^	Diameter ca. 40 nm	Kud: Hr-hairless mice exposed to intense UVB (302 nm); RNP^N^ 300 mg/kg (total) given in the drinking water (0.5 mg/mL) for 37 d	Significant reduction in UVB-induced skin aging, epidermal thickening, edema, erythema and skin lesions	[193]

## Data Availability

Not applicable.

## Abbreviations

6-OHDA	6-hydroxydopamine
A2E	bis-retinoid N-retinyl-N-retinylidene ethanolamine (A2E), a major component of lipofuscin in the retina
AMD	age-related macular degeneration
Asc	ascorbate
t-BHP	*tert*-butyl hydroperoxide
BSA	bovine serum albumin
But	butyl
DTNB	5,5-dithio-bis-(2-nitrobenzoic acid) (Ellman’s reagent)
EPR	electron paramagnetic resonance
GLUT	glucose transporter
GSH	glutathione
HO-1	heme oxygenase 1
IC_50_	half-inhibitory concentration
IL	interleukin
IR	ionizing radiation
LDH	lactate dehydrogenase
MMP	matrix metalloproteinase
mtDNA	mitochondrial DNA
NHE	normal hydrogen electrode
NMR	nuclear magnetic resonance
pCMB	*para*-chloromercuribenzoate
PEG	poly(ethylene glycol)
PMOT	poly [4-(2,2,6,6-tetramethylpiperidine-1-oxyl)oxymethylstyrene]
PROXYL	2,2,5,5-tetramethylpyrrolidinyl-1-oxyl
RNP	redox nanoparticles
ROS	reactive oxygen species
SOD	superoxide dismutase
TBARS	thiobarbituric-acid reactive substances
TEMPO	2,2,6,6-tetramethylpiperidine-1-oxyl
TEMPAMINE	4-amino-2,2,6,6-tetramethylpiperidine-1-oxyl
TEMPOL	4-hydroxyl-2,2,6,6-tetramethylpiperidine-1-oxyl
TEMPOL-H	hydroxylamine of TEMPOL
